# Early life nicotine vapor exposure increases nicotine vapor seeking in adult male rats

**DOI:** 10.1007/s00213-026-07122-8

**Published:** 2026-07-14

**Authors:** Liliana Maynez-Anchondo, Olga Rohrer, Miguel Urbina, Jocelyn Aguilar Armenta, Tiffany Gonzalez-Gutierrez, Laura E. O’Dell, Ian A. Mendez

**Affiliations:** 1 Department of Psychology, The University of Texas, El Paso, TX, USA; 2 School of Pharmacy, The University of Texas at El Paso, 1101 N. Campbell Street Room #110, El Paso, TX 79902, USA

**Keywords:** Nicotine, Vapor, Electronic Cigarettes, Adolescence, Motivation, Self-Administration, Rats

## Abstract

**Rationale:**

The ongoing epidemic of nicotine vaping use in adolescents and young adults presents a significant health concern, yet the long-term effects of nicotine vaping remain largely unknown.

**Objective:**

The present study aimed to investigate how nicotine vapor intake early in life influences reward-seeking behavior immediately after exposure and later in adulthood.

**Methods:**

Experiment 1 was completed in 3 phases. In Phase 1, 24 male adolescent Sprague-Dawley rats (PND = 58) were passively exposed to vehicle (propylene glycol/vegetable glycerin, 50/50) or nicotine (24 mg/mL) vapor and immediately tested in a Progressive Ratio (PR) task that utilized food pellet delivery as reinforcement for 10 consecutive days. In Phase 2, the PR testing was replicated in early adulthood (PND = 140), with groups further divided into four groups: No exposure, adolescent exposure, early adulthood exposure, and both adolescent and early adulthood exposure. Lastly, in Phase 3, during late adulthood (PND = 341), all four vapor groups underwent nicotine vapor self-administration (NVSA) testing (6 mg/mL). For Experiment 2, 16 male young adult Wistar rats received vehicle or nicotine vapor deliveries during the PR task. Rats were given access to increasing concentrations of nicotine (1, 3, 6, 12, 24 mg/mL) for two days at each concentration. After the PR tests, the rats were given two additional days of NVSA (24 mg/mL) on a Fixed Ratio 1 (FR1) schedule of reinforcement.

**Results:**

Experiment 1 revealed that adolescent but not adult exposure to 24 mg/mL of nicotine vapor, resulted in an increase in both active and inactive lever presses relative to controls. Notably, rats in the adolescent and early adulthood exposure groups displayed significant increases in active but not inactive port nosepokes later in life, when compared to rats that were not previously exposed to nicotine vapor. Experiment 2 revealed no group differences in responding for vapor deliveries in the PR task when testing young adult rats. However, there was more active port nosepoking compared to vehicle vapor controls, when testing using an FR1 schedule of reinforcement.

**Conclusions:**

These results demonstrate that exposure to nicotine vapor early in life may increase nicotine seeking behavior in adulthood, suggesting that restricting early e-cigarette use may reduce future nicotine intake and the negative health consequences associated with its use.

## Introduction

Nicotine is a highly addictive substance that affects the brain’s reward system and can lead to dependency and withdrawal. In the United States, nicotine use is the leading preventable cause of disease, death, and disability, with approximately 480,000 fatalities attributed to nicotine dependence in 2024 (Center for Disease Control and Prevention, 2023). Additionally, over $241 billion was spent on health costs related to nicotine use in 2018 (Center for Disease Control and Prevention 2024). Studies show that only about 5–7% of nicotine users successfully quit without assistance each year, and most people attempt to quit multiple times before succeeding (Caldeira et al. 2012; Chaiton et al. 2016; Fiore et al. 2008). Chronic nicotine use impairs global health, with negative effects commonly observed on cardiovascular, pulmonary, and metabolic functions (Benowitz 2010; Cruz et al. 2021; Oakes et al. 2020). The neurotoxic effects of nicotine are particularly concerning, as they disrupt brain development, leading to cognitive impairments and a heightened risk of substance use disorder (Goriounova and Mansvelder 2012).

Electronic cigarettes (i.e., e-cigarettes) were introduced to the US market in 2007 as a harm-reduction alternative to smoking and as a smoking cessation aid. However, recreational vaping appeals not only to smokers but also to non-smokers (Cummings and Proctor 2014; Wang et al. 2021). While educational campaigns have been effective in reducing the use of traditional cigarettes for many years, the arrival of e-cigarettes has created a new crisis of nicotine dependence, primarily among adolescents and young adults (Flores et al. 2021; Gentzke et al. 2022). Nicotine exposure during adolescence, a critical period of brain development, has detrimental effects on health and behavior. Specifically, research has shown that early exposure to drugs of abuse can disrupt normal brain maturation in areas that control reward processing, impulse control, motivation, cognition, and executive functions (Leslie 2020; Mahajan et al. 2021; Yuan et al. 2015). Remarkably, the 2022 National Youth Tobacco Survey (NYTS) found that 27.2% of middle and high schoolers who consume nicotine reported low academic performance, while 18.3% suffered severe symptoms of psychological distress. Furthermore, clinical research has found that adolescent e-cigarette use is associated with a greater risk of future combustible cigarette smoking (Epstein et al. 2021; O’Brien et al. 2021).

Passive exposure to nicotine vapor has been utilized as a method for establishing nicotine dependence in rodents, modeling e-cigarette vapor exposure in humans. Rodents exposed to nicotine vapor in this model demonstrate increased levels of nicotine and cotinine in their blood plasma at levels comparable to those observed in human e-cigarette users (Flores et al. 2021; Montanari et al. 2020).

They also exhibited nicotine-induced conditioned place preference (Frie et al. 2020), hyperalgesia (Baiamonte et al. 2014; Chellian et al. 2021), spontaneous and drug-precipitated withdrawal (Martinez et al. 2023; Montanari et al. 2020), changes in intracranial self-stimulation thresholds (Henderson and Cooper 2021), increases in intravenous nicotine intake (Gilpin et al. 2014; Kallupi et al. 2019), and short-term increases in impulsive and risky choice (Flores et al. 2021; Giner et al. 2022), with some studies highlighting sex-dependent changes in behavior and brain function (Espinoza et al. 2022; Lallai et al. 2021). The present study also utilizes a nicotine vapor self-administration (NVSA) rodent model. NVSA models in rats have demonstrated nicotine dependence-like behaviors, withdrawal symptoms, psychomotor stimulation, and blood cotinine levels comparable to intravenous nicotine self-administration (Lallai et al. 2021; Smith et al. 2020). An NVSA model in rats offers greater translatability to human nicotine use because, unlike traditional nicotine delivery methods such as injections or minipumps, vapor inhalation allows the subject to experience the distinct sensations and effects associated with nicotine absorption through the respiratory system. Additionally, like other self-administration models, NVSA models capture volitional aspects of nicotine intake, making them more relevant to nicotine use in humans (LeSage et al. 2003; O’Dell et al. 2007; Smith et al. 2020).

For the 11th consecutive year, e-cigarettes remain the most used form of nicotine (Center for Disease Control and Prevention, 2023). Alarmingly, the use of e-cigarettes among adolescents sharply increased from 2010 to 2017, leading the U.S. to declare an epidemic of youth nicotine vaping (Gottlieb 2018). While e-cigarettes are relatively novel and the effects of inhaling vapors containing nicotine and other compounds are not well understood, many of the adolescents who initiated e-cigarette use during their teens, are continuing to vape well into early adulthood, and even parenthood. Studies over the last 10 years, from our lab and others, have begun to identify potential long-term health risks of vaping (Giner et al. 2022; Kaisar et al. 2016; Lerner et al. 2015). Given the ongoing vaping epidemic, and the well described health risks associated with nicotine use, the present study aimed to assess how early life exposure to nicotine vapor can influence nicotine abuse liability immediately after use and later in life.

## Methods

### Subjects

For Experiment 1, 24 male Sprague-Dawley rats were obtained from an outbred stock of animals (Envigo, Inc., Indianapolis, IN, USA) that arrived in the vivarium at postnatal day (PND) 25. For Experiment 2, 16 male Wistar rats were bred in-house from an outbred stock (Envigo, Inc., Indianapolis, IN, USA) and began described training procedures at PND 86. All rats were paired-housed in a humidity and temperature controlled (22 °C) vivarium on a standard 12-hour light/dark cycle (lights off at 8:00 pm, on at 8:00 am) with ad libitum access to food and water, except during behavioral training and testing. All animal procedures were approved by the University of Texas at El Paso Institutional Animal Care and Use Committee and are in compliance with the Guide for the Care and Use of Animals (National Research Council 2012).

### Drugs

For both Experiment 1 and 2, flavorless e-liquids were purchased from a commercial vendor with nicotine prepared in its freebase form in a vehicle solution of 50% propylene glycol and 50% vegetable glycerin (Vapor Vapes, Sand City, CA, USA). For passive exposure e-liquids containing 0 (i.e., vehicle vapor) or 24 mg/mL of nicotine were used. For self-administration of vapor, nicotine e-liquid with a concentration of 6 mg/mL nicotine was used. Experiment 2 utilized e-liquids with concentrations of 0, 1, 3, 6, 12, or 24 mg/mL nicotine for vapor deliveries in the PR task and NVSA.

### Apparatus

#### Chambers for nicotine delivery and exposure with NVSA and nicotine-based PR task

The subjects were passively exposed to vapor using a four chamber Quad Passive Vapor Inhalation System (La Jolla Alcohol Research Inc., La Jolla, CA, USA). The four airtight chambers had interior dimensions of 14.5” L x 10.5” W x 9.0” H. Each chamber had an output valve port that was connected via PVC tubing to an external vacuum that produced negative pressure in the chamber with an airflow at 0.6 L per minute. The vacuum outlet was connected to a Whatman HEPA filter (Millipore Sigma, Darmstadt, Germany) and onto a building exhaust system that removed the vapor from the chambers. At the opposite wall where the output valve port was located, there was the input valve port, it was connected via PVC tubing to a TFV4 cartridge (4.9-volt, 65.0 W: Smok Inc., Shenzhen, China), where the nicotine e-liquid was heated by a 0.42 Ω atomizer coil (Flores et al. 2021; Giner et al. 2022).

NVSA was conducted using four transparent Plexiglas sealed chambers and the Quad Self-Administration Vapor Inhalation System (14 cm × 20 cm × 23 cm; La Jolla Alcohol Research, La Jolla, CA, USA). Active and inactive nosepoke holes were mounted in the vapor exposure chambers opposite each other on the side walls 6.5 cm from the back wall. A white cue light was located directly above each nosepoke hole, and both holes and cue lights were assigned as active and inactive in a counterbalanced manner. The top of each exposure chamber had an output valve port that was connected via PVC tubing to a house vacuum. The vacuum outlet was connected to a Whatman HEPA filter (Millipore Sigma, Darmstadt, Germany) and to a building exhaust system that removed the vapor from the chambers. Airflow rate in the chambers was controlled with an external airflow gauge connected to each chamber and the experiment was conducted with an airflow rate of 1 L per minute (Marchette et al. 2021; Moussawi et al. 2020). The output valve port was located on the left wall of each chamber, 9 cm from the front wall. The input valve port, it was connected via PVC tubing to a TFV4 cartridge (4.9-volt, 65.0 W: Smok Inc., Shenzhen, China), where the nicotine e-liquid was heated by a 0.42 Ω atomizer coil (Flores et al. 2021; Giner et al. 2022). Both active and inactive cue lights and nosepoke holes in the self-administration system were connected to a Med-Associates SmartCtrl^™^ Connection Panel, which then connected to a computer with Med PC-V (Med Associates, Fairfax, VT, USA).

#### Operant boxes used for food-based PR task

Eight standard operant chambers (Med Associates, Fairfax, VT, USA) were used to train and test the subjects in the PR task. Each chamber (31 × 25 × 31 cm) was individually nested in a sound-attenuating cubicle. The chamber front wall was made of aluminum, it had a recessed food trough (4.1 × 3.2 cm) into which the food pellet rewards were delivered, and a sensor was able to detect and register head entries. A 1.12-watt light was at the center of the wall, above the food trough. A retractable response lever was located at each side of the food trough. The back, ceiling and the side walls of the chamber were made of acrylic while the floor was made of steel rods (Giner et al. 2022; Mendez et al. 2013). Behavioral responses were automatically recorded by a computer using Med PC-V (Med Associates, Fairfax, VT, USA).

### Experimental procedures for Experiment 1

Procedures for Experiment 1 were divided into 3 phases as described below. Major experimental procedures across all phases are visually summarized in Fig. 1.

Schematic showing nicotine vapor exposure and testing across experiment phases for Experiment 1.

#### Phase 1: Adolescent passive vapor exposure and testing in food-based PR task

Prior to the behavioral training, rats were food restricted to 90% of their free feeding weight (FFW) and weighed every other day to maintain target weights. Rats were fed 5–12 g per day based on the target weight, with weight gain allowances estimated weekly based on their age and a standard growth curve for Sprague-Dawley rats that was provided by Envigo Inc., in addition to the 45 mg grain food pellets earned during training and testing in the PR task (PR). The experiment schedule began with the rats aged PND 31. The subjects went through shaping procedures for the PR task, which included one day of magazine training, three days of lever press training for food pellets, and six days of instrumental training with ascending random interval schedules between days (15, 15, 30, 30, 60, 60 s). For a detailed description of the food magazine shaping and lever press training procedures for the PR task see previous work from our laboratory (Mendez et al. 2010). Assignment of active and inactive levers was counterbalanced between the left and right levers, and the criteria for acquisition of lever press training was 30 active lever presses in 30 min or less. After training procedures, rats were trained to respond for food pellets in a 1-hour PR testing session. For this food-based progressive ratio task the number of lever presses required to receive 2 food pellets progressively increased by 1 response for each subsequent reward (i.e., 1, 2, 4, 7, 11, etc.).

Rats were trained in the PR task for five days before beginning passive exposure to vehicle vapor immediately prior to training. A passive nicotine vapor exposure session consisted of four cycles of 18 min and 30 s, with 5-minute inter-cycle intervals. For each cycle, the e-juice was heated to 400° F for a 3-second puff delivery, occurring every two minutes and ten times per cycle. The sessions lasted 89 min and 30 s and delivered 40 nicotine puffs. After 12 days of exposure to the vehicle vapor followed by PR testing, rats were divided into two groups of 12 rats each (PND 58). One group continued to be passively exposed to vehicle vapor, followed immediately by testing in the PR task. The other group was passively exposed to 24 mg/mL of nicotine vapor, before testing in the PR task. Both groups were tested for 10 consecutive days in this first phase of the experiment, finishing at PND 67. PR testing continued for 3 days after cessation of vapor exposure.

#### Phase 2: Early adulthood passive vapor exposure and testing in food-based PR task

Twenty-eight days after adolescent vapor exposure, rats had transitioned into early adulthood (PND 95) and the effects of a single nicotine vapor exposure on PR performance were assessed using the same PR parameters described for the adolescent test. For this single day nicotine challenge, the groups of 24 rats previously exposed to vehicle or 24 mg/mL nicotine vapor were further divided into two more groups. Half of the PG/VG and half of the 24 mg/mL adolescent exposure groups were passively exposed to vehicle vapor, while the other half of these groups were exposed to 24 mg/mL nicotine vapor. This resulted in a total of 4 groups, with 6 rats per group: No nicotine exposure during adolescence or adulthood (0–0), adolescence nicotine exposure only (24–0), early adulthood nicotine exposure only (0–24), and both adolescence and early adulthood nicotine exposure (24–24).

After this single test, rats were returned to their home cages for an additional 45 days, before being tested in the PR task following repeated vapor exposure, again in early adulthood (PND 140). The experimental testing from Phase 1 was replicated, with a few minor differences. For this test, rats were habituated to vehicle vapor for 20 consecutive days before half were exposed to nicotine vapor (PND 160). Additionally, this early adulthood test of repeated exposure to vehicle or nicotine vapor continued to use the 4 treatment groups described for the single nicotine vapor challenge described above (no nicotine vapor exposure, adolescence nicotine exposure only, early adulthood nicotine exposure only, and both adolescence and early adulthood nicotine exposure). As before, rats were tested in the PR task immediately following passive exposure to vehicle or 24 mg/mL nicotine vapor for 10 consecutive days (PND 159–168) and again followed by 3 days of post-vapor PR testing.

#### Phase 3: Adulthood testing in NVSA task

In the last part of the experiment, during late adulthood, all four passive vapor treatment groups used in Phase 2 were trained and tested for NVSA using a nicotine concentration of 6 mg/mL. Rats were exposed daily to 1 h session that consisted of a one-second nicotine vapor plume delivered following a nosepoke in the active aperture, using a fixed ratio 1 (FR1) schedule with a 2-minute time-out period after each vapor delivery. The criterion for acquisition of NVSA was met when all rats exhibited less than 15% variability in average rewards received across two consecutive days (Belluzzi et al. 2004). Acquisition was achieved for all groups within 6 days (PND 341–346), after which subjects were tested in the task for 4 additional days (PND 347–350).

### Experimental procedures for Experiment 2

#### Early adulthood testing in nicotine-based PR task

For Experiment 2, rats were trained and tested in a PR task using the same procedures and PR response requirements described for Phase 1 and 2 of Experiment 1. However, instead of responding for food pellet deliveries, behavioral responding was reinforced with deliveries of vehicle or nicotine vapor plumes. Following 12 days of training in the PR task with delivery of vehicle vapor, rats were split into two groups, with eight rats per group. One group continued to respond for vehicle vapor throughout all tests assessing responding in the PR task and the other group responding for nicotine vapor in the PR task. Rats were tested for 10 days in the PR task with concentrations of nicotine that increased every 2 days (1, 3, 6, 12, 24 mg/mL).

#### Early adulthood testing in NVSA task

Following testing in the PR task, rats were tested for two additional days in the NVSA task as described above in Phase 3 of Experiment 1. However, for this experiment, rats responded for 24 mg/mL nicotine vapor deliveries instead of food pellet deliveries. Additionally, rats were not food-deprived at any point, daily test sessions were 3 h in length, and a 2-secont time-out period was activated immediately after each vapor delivery.

### Statistics

To assess the effects of nicotine vapor exposure to increase food reward-seeking behavior in the PR task, total lever presses were determined, and the percent change from baseline was analyzed for each group using repeated-measures ANOVA. Differences between treatment groups were subsequently compared using a paired samples t-test (Phase 1) or Tukey’s LSD (Phase 2). Additionally, a discrimination index (i.e., ratio of active to inactive lever presses) was calculated for each PR test day (active lever presses – inactive lever presses/total lever presses) and compared using independent samples t-tests. To determine the effects of passive exposure to nicotine early in life on nicotine vapor seeking in late adulthood, total nicotine vapor plume deliveries (i.e., rewards received), active port nosepokes (i.e., reward-seeking nosepokes), and inactive port nosepokes were collected and analyzed using repeated-measures ANOVA, which was followed by Fisher’s LSD or one-way ANOVAs to determine specific group differences on our measures of interest during test days (Phase 3). For Experiment 2, the average of two days of testing with each concentration of nicotine tested was taken and used to calculate percent change from baseline. Partial eta squared and Cohen’s d were calculated to determine the effect size of our main effects and interactions.

## Results

### Experiment 1, Phase 1: Adolescent exposure to nicotine vapor and testing in the PR task

Evaluation of descriptive statistics for active lever presses during the 10 days of passive vapor exposure in adolescents revealed one rat in the vehicle vapor group that was more than two standard deviations outside the group mean on vapor exposure days 4–10. This rat was excluded from the study. Data reveals that all rats pressed more for the active lever (Ave: 709.38, SEM:140.41) than they did for the inactive lever (Ave: 31.22, SEM: 8.58), across baseline testing in the PR task (i.e., the last three days of vehicle vapor exposure). Importantly, a comparison of average number of lever presses across baseline testing days in the PR task showed no significant differences in the number of active (*F*_(1,22)_=0.01, *p*=0.98) or inactive (*F*_(1,22)_=1.24, *p*=0.28) lever presses, when comparing rats that continued to receive vehicle vapor to those that transitioned to nicotine vapor exposure.

During the PR task that immediately followed nicotine vapor exposure, rats continued to press more on the active lever that delivered food pellets (Ave: 811.55, SEM:127.04) as compared to the inactive lever (Ave: 39.72, SEM: 9.40). Repeated measures ANOVA comparing percent change from baseline in active lever presses between treatment groups, across 10 days of adolescent vapor exposure, revealed a significant main effect of day (*F*_(9,180)_=6.56, *p*<0.001, $\eta_{p}^{2}=\;0.25$), but no main effect of group across all 10 days (*F*_(1,20)_=3.32, *p*=0.08). Analysis also revealed a significant interaction of treatment day and group (*F*_(9,180)_=6.56, *p*<0.001, $\eta_{p}^{2}=\;0.14$), such that an observed increase in total lever presses in the nicotine vapor group relative to controls decreased across treatment days (Fig. 2a). Post-hoc analysis comparing percent change in active lever presses between the two vapor groups on each day of treatment show that the group exposed to nicotine vapor had significantly more active lever presses on days 1, 5, and 7 (*p_s_*<0.05, *Cohen’s d*>−0.71).

Repeated measures ANOVA comparing precent change from baseline in inactive lever presses between treatment groups across 10 days of adolescent vapor exposure also revealed a significant main effect of day (*F*_(9,189)_=3.74, *p*<0.001, $\eta_{p}^{2}=\;0.15$). In the case of inactive lever presses no significant interaction of treatment day and group was seen (*F*_(9,189)_=1.29, *p*=0.25), but a main effect of treatment group was (*F*_(1,21)_=5.37, *p*<0.05, $\eta_{p}^{2}=\;0.204$), such that animals exposed to nicotine vapor had more inactive lever presses than vehicle controls (Fig. 2b). Post-hoc analysis comparing percent change in inactive lever presses between the two vapor groups on each day of treatment show that the group exposed to nicotine vapor had significantly more inactive lever presses on all treatment days, excluding day 8 (*p_s_*<0.05, *Cohen’s d*>−0.77). To better assess the effect of nicotine on inactive lever presses, an active to inactive lever press discrimination index was calculated for each rat in the PR task. A comparison of this index between vehicle and nicotine vapor exposed rats revealed no significant differences between the groups on any of the vapor exposure test days (*t_s_*<1.02, *p_s_*>0.16). Percent change from vapor exposure days was also calculated and assessed for 3 consecutive days following passive vapor exposure; however, no significant main effects or interactions were observed for either active (*F_s_*<2.36, *p_s_*>0.12) or inactive (*F_s_*<3.89, *p_s_*>0.06) lever presses.

### Experiment 1, Phase 2: Early adulthood exposure to nicotine vapor and testing in the PR task

Following adolescent vapor exposure, rats were allowed to transition into early adulthood (PND 95) and the effects of a single passive nicotine vapor exposure on lever presses in the PR task were assessed. As previously described in the methods section, each of the 2 treatment groups used for adolescent exposure (vehicle or 24 mg/mL nicotine vapor) was further divided, with half of each group receiving vehicle or nicotine vapor during early adolescence, resulting in 4 groups (no nicotine vapor exposure, adolescence-only nicotine vapor exposure, early adulthood-only vapor exposure, and adolescent and early adulthood nicotine vapor exposure). Analysis of this single exposure using one-way ANOVAs revealed no significant main effect of group for active (*F*_(322)_=0.03, *p*=0.99) or inactive lever presses (*F*(3,20)=0.4^,^5, *p*=0.72).

Sixty-five days after the single exposure test (PND140), the effects of repeated passive nicotine vapor exposure on lever presses in the PR task were again assessed. Treatment groups were identical to those used during the single exposure test. Repeated measures ANOVA comparing active lever presses between treatment groups, across 10 days of early adulthood vapor exposure, revealed a significant main effect of day (*F*_(9,171)_=3.12, *p*<0.01, $\eta_{p}^{2}=\;0.14$), but no main effect of group across all 10 days (*F*_(3,19)_=0.06, *p*=0.88). Analysis again revealed a significant interaction of treatment day and group (*F*_(27,171)_=2.11, *p*<0.01, $\eta_{p}^{2}=\;0.25$). While a significant interaction of treatment day and group was observed, one-way ANOVAs comparing active lever presses between the four vapor groups on each day of treatment show that active lever presses did not differ between groups on any given day (F*_s_*<0.59, p*_s_*>0.62).

Repeated measures ANOVA comparing inactive lever presses between treatment groups, across 10 days of early adulthood vapor exposure, also revealed a significant main effect of day (*F*_(9,180)_=2.86, *p*<0.01, $\eta_{p}^{2}=\;0.13$), but no main effect of group across all 10 days (*F*_(3,20)_=1.71, *p*=0.20) or interaction of treatment day and group (*F*_(27,180)_=0.92, *p*<0.59). As with adolescent exposure, lever presses were assessed for 3 consecutive days following early adulthood vapor exposure. Analysis again revealed no significant main effects or interactions for active (*F_s_*<1.35, *p_s_*>0.27) and inactive (*F_s_*<1.93, *p_s_*>0.16) lever presses in the PR task.

### Experiment 1, Phase 3: Adulthood NVSA

All four groups used in Phase 2 (no nicotine exposure, nicotine adolescent exposure only, nicotine early adulthood exposure only, nicotine adolescence and early adulthood exposure) received access to NVSA later in adulthood (PND 341). Criteria for stability in the NVSA task was met after 6 days of training in NVSA. NVSA Training days 1–6 were analyzed with repeated measures ANOVA and resulted in no significant main effect of group or interactions of group and day when comparing number of plumes delivered (*F_s_*<0.45, *p_s_*>0.72) total active port nosepokes (*F_s_*<0.89, *p_s_*>0.46), and total inactive port nosepokes (*F_s_*<1.61, *p_s_*>0.08). There was, however, an expected significant main effect of NVSA day seen for all measures assessed across NVSA days 1–6 (*F_s_*>5.82, *p_s_*<0.001,$\eta_{p}^{2}>0.24$), such that plume deliveries (Fig. 3a), active port pokes (Fig. 3c), and inactive port pokes increased across training days in the task.

The effects of adolescent and early adulthood exposure on nicotine seeking later in life were assessed across days 7–10 of NVSA using repeated measures ANOVA. Analysis revealed no main effect of group (*F*_(3,19)_=2.50, *p*=0.09), main effect of day (*F*_(3,57)_=1.88, *p*=0.14), or interaction of group and day (*F*_(9,57)_=4.88, *p*=0.43) for total plumes delivered across NVSA days 7–10 (Fig. 3b). While analysis of active port nosepokes did not result in a significant interaction of group and day (*F*_(3,20)_=0.45, *p*=0.72), a main effect of NVSA day (*F*_(3,57)_=4.17, *p*<0.05, $\eta_{p}^{2}=\;0.18$) did emerge, such that active port nosepokes slightly decreased across day 7–10 (Fig. 3d). Interestingly, statistical analysis of total active port nosepokes across NVSA days 7–10 did result a main effect of treatment group (*F*_(3,19)_=3.31, *p*<0.05, $\eta_{p}^{2}=\;0.34$). Fisher’s LSD was used to compare groups across days 7–10 and demonstrated that rats exposed to nicotine vapor during adolescence or early adulthood, but not both, had more active port nosepokes relative to rats with no early life exposure to nicotine vapor (*p*_s_<0.05). Nosepokes in the inactive port were also assessed across days 7–10 using one-way ANOVA, however no main effects or interactions were observed for this measure (*F_s_*<1.61, *p_s_* > 0.36).

### Experiment 2: Early adulthood testing in PR task utilizing vapor delivery and NVSA behavior

Analyses of nosepoke responses in a PR task that delivered vapor reveal that all rats had a similar nosepokes in the active port (Ave: 25.10, SEM:5.61) to those seen for the inactive port (Ave: 25.38, SEM: 5.19), across baseline testing in the task (i.e., the last three days of vehicle exposure). Importantly, a comparison of average number of nosepokes across baseline testing days in the PR task delivering nicotine vapor showed no significant differences in the number of active (*F*_(1,14)_=0.01, *p*=0.93) or inactive (*F*_(1,14)_=1.98, *p*=0.18) port nosepokes, when comparing rats that continued to receive vehicle vapor to those that transitioned to nicotine vapor exposure.

Repeated measures ANOVA analyzing percent change from baseline in active port nosepokes for 5 concentrations of nicotine vapor (averaged across of two days of testing with each concentration) revealed a significant interaction of concentration tested and treatment (*F*_(4,56)_=14.69, *p*<0.01, $\eta_{p}^{2}=\;0.51$), such that tests utilizing lower concentration of nicotine decreased active port nosepokes relative to baseline, while higher concentration nicotine and vehicle vapor did not shift active port nosepokes relative to baseline (Fig. 4a). No main effects of treatment or concentration were observed. Post-hoc analysis comparing percent change in active port nosepokes between the two treatment groups at each concentration show that young adult rats responding for nicotine had fewer active port nosepokes for lower concentrations (1 and 3 mg/mL) and more active port nosepokes for the highest concentration (24 mg/mL), relative to rats responding for vehicle vapor (*p_s_*<0.05, *Cohen’s d*>1.09). Repeated measures ANOVA analyzing percent change from baseline in inactive port nosepokes for 5 concentrations of nicotine vapor (averaged across of two days of testing with each concentration) revealed no significant main effects of treatment (*Fs*_(1,14)_=0.19, *p*=0.67) or concentration (*Fs*_(4,52)_=0.92, *p*=0.46). Similarly, no interaction (*F*_(4,54)_=0.05, *p*=0.82) of treatment or concentration was observed (Fig. 4b).

Immediately following tests of nicotine seeking behavior in the PR task, nicotine seeking was tested in two daily NVSA sessions that were 3 h in length and delivered nicotine on an FR1 schedule of reinforcement. Independent samples t-test comparing active port nosepokes between rats pressing for vehicle vapor and rats pressing for 24 mg/mL nicotine vapor revealed a significant main effect of treatment (*t*=2.57, *p*_s_=0.01, *Cohen’s d*=1.28), such that rats responding for nicotine had significantly more active port nosepokes than vehicle controls (Fig. 4c). Analysis of inactive port nosepokes revealed no significant differences between rats pressing for vehicle vapor and rats pressing for 24 mg/mL nicotine vapor (*t*=0.02, *p*_s_=0.49).

## Discussion

Adolescence is a crucial period of brain development when individuals are vulnerable to the maladaptive neuroplasticity that follows nicotine exposure(Castro et al. 2023; Goriounova and Mansvelder 2012; Yuan et al. 2015). In the present study, we evaluated the impact of nicotine vapor exposure during adolescence and/or early adulthood on both short- and long-term reward-seeking behavior (Experiment 1). During this experiment’s first phase, male adolescent rats were passively exposed to nicotine vapor and immediately tested in the food-based PR task. Although cotinine levels were not measured in the current experiment, the passive exposure procedures used were identical to our laboratories established paradigm, which we have previously shown to significantly increase cotinine levels similar to that seen in human e-cigarette users (Flores et al. 2021; Giner et al. 2022). Analysis of behavioral responding in the PR task reveal that rats with nicotine vapor exposure exhibited more active lever presses for food reward in the PR task, compared to rats in the vehicle control group. Importantly, increases in inactive lever presses were also observed, suggesting that nicotine vapor may increase general activity, arousal, or nonspecific responding rather than selectively increasing food-directed motivation. The second phase of this experiment replicated vapor exposure and PR testing in Phase 1 but was conducted during early adulthood. Groups were further divided for this phase, resulting in a total of four nicotine exposure groups: no nicotine exposure, adolescent nicotine exposure, early adulthood nicotine exposure, and adolescence and early adulthood nicotine exposure. Testing in the PR task following exposure in Phase 2 resulted in no significant group differences in lever presses for food rewards, suggesting that nicotine’s ability to cause immediate increases in motivation for reward is more robust in adolescence versus early adulthood.

The third phase of this experiment was conducted during late adulthood and tested the four groups from phase two in the NVSA task (6 mg/ml nicotine vapor). With our NVSA model, it took the rats 6 days of training to stabilize their responding in the task, which is similar to that seen with another NVSA model recently described (Smith et al. 2020). Testing in our study revealed that relative to rats that were not exposed to nicotine vapor early in life, rats exposed to nicotine vapor during adolescence or early adulthood exhibited a significant increase in active port responding, but not the inactive port. Interestingly, rats that were exposed to nicotine vapor during both adolescence and early adulthood did not show significant increases in active port nosepokes, suggesting that the observed long-term effects may only occur under specific conditions. Furthermore, no differences in nicotine vapor plume deliveries were observed. This implies that while early life nicotine vapor exposure may increase nicotine seeking behaviors, it may not necessarily increase nicotine vapor self-administration. Together these results demonstrate that exposure to nicotine vapor during the developmental stage of life can escalate nicotine-seeking well into adulthood, despite extended periods of abstinence. These findings align with clinical studies that have found the use of e-cigarettes among youths increased the risk of nicotine use later in life (Berry et al. 2019; Evans-Polce et al. 2024; Meehan et al. 2024). However, additional studies will be required to determine whether early life nicotine vapor exposure increases nicotine vapor self-administration and vulnerability for nicotine use disorder later in life.

There was concern that the effects of food-deprivation and the appetite suppressant effects of nicotine could present a confound in the PR task given that food was used as a rein-forcer in Experiment 1. Therefore, the PR task was repeated using nicotine vapor deliveries in young adult rats (Experiment 2). Findings from this study revealed no significant increases in active port nosepokes in rats responding for 1, 3, 6, or 12 mg/mL nicotine, relative to those responding for vehicle vapor. Rats responding for 24 mg/mL in the PR task did have significantly more nosepokes in the active port relative to controls; however, responding for this concentration of nicotine resulted in a similar number of responses to that seen during baseline measures, suggesting that this difference was a result of a decrease in responding for vehicle vapor, rather than an increase in nicotine seeking behavior. Following testing in the PR task that delivered nicotine vapor, rats were tested for NVSA on an FR1 schedule. Analysis of responding in this task revealed more active port nosepokes in rats responding to 24 mg/mL nicotine, relative to rats responding to vehicle vapor. Together these findings indicate that nicotine vapor induces immediate reinforcement of behavior than vehicle control and better discrimination between active and inactive ports when using FR1 schedules of reinforcement relative to PR schedules. The low level of nicotine vapor seeking behavior in our PR task is consistent with previous studies using intravenous nicotine self-administration (Donny et al. 1999). Also, responding for nicotine delivery in the PR task appears to be less robust than PR responding for other stimulant drugs such cocaine (Richardson and Roberts 1996) and amphetamine (Mendez et al. 2009). Recent studies with mice also reveal that more nicotine vapor deliveries occur when responding under fixed ratio schedules of reinforcement, relative to progressive ratio schedules (Cooper et al. 2023, 2024).

Cue-induced increases in nicotine seeking and taking that extend beyond the initial period of withdrawal have been documented in humans and animals (Pickens et al. 2011). This protracted craving of nicotine, often referred to as incubation, is believed to drive relapse in humans, even after months of abstinence (Bedi et al. 2012; Kota et al. 2009). While our findings are the first to demonstrate that early life nicotine exposure increases nicotine vapor-seeking later in life, previous animal studies have demonstrated that nicotine injections during adolescence can increase sensitivity to nicotine in adulthood. For example, it has been shown that adult rats exposed to nicotine injections during adolescence display a heightened nicotine-induced locomotor stimulant effect (Bracken et al. 2011) and an increase in cue-induced nicotine seeking and self-administration (Adriani et al. 2003; Markou et al. 2019; Renda et al. 2020). Similarly, early adolescent exposure to nicotine in mice results in increased nicotine reward as determined by the conditioned place preference task (Kota et al. 2009). This study in mice also revealed that adolescent exposure, but not adult exposure to nicotine, caused an increase in nicotinic receptor function in the cortex, striatum, and hippocampus 7-weeks after exposure, relative to age-matched saline controls. Studies in rats have also identified possible neurobiological mechanisms driving long-term incubation of nicotine craving, such as increases in α5, α6, and β2 nicotinic acetylcholine receptor (nAChR) subunit gene expression in the ventral midbrain 5 weeks after adolescent exposure to nicotine (Adriani et al. 2003). Interestingly, more recent studies, including one from our laboratory, have also identified long-term decreases in β2 nAChR subunit gene expression within the nucleus accumbens, 4 and 7 weeks after exposure to nicotine vapor in adult male rats (Giner et al. 2022; Smith et al. 2020).

While behavioral and neurobiological effects observed following early-life exposure to nicotine have been observed, the effects that are unique to this stage of development remain fully understood. Findings from our study do indicate that early-life nicotine exposure significantly increases nicotine-seeking behavior later in life, but due to the absence of an adult treatment group, we cannot determine if this outcome is distinct to the malleable adolescent brain. This represents a limitation for the described work and highlights the importance of including adult treatment groups in future studies aimed at identifying effects of nicotine exposure that are distinct to the developing brain. Another methodological limitation of the present study lies in the whole-body exposure that occurred with our passive and NVSA exposure models, as transdermal and gastrointestinal absorption may occur. This reduces control of systemic absorption and increases variability of measured outcomes. Additionally, the absence of female rats also represents a constraint in our design. Including female rats in pre-clinical studies investigating nicotine seeking behavior is vital, as sex differences in the mechanisms driving nicotine use disorder have been well documented. Female rats display higher sensitivity to the rewarding effects of nicotine and stronger withdrawal symptoms compared to males, a finding that is consistent with human nicotine use patterns (Flores et al. 2019; Giner et al. 2024; Lallai et al. 2021). It is acknowledged that the exclusion of female rats in the present study represents a limitation of this work, especially because including both sexes ensures that experiments capture the nuances of nicotine use in the human population and closes the health disparities that have resulted from the historic use of predominantly male subjects in pre-clinical research. We therefore emphasize the need for future studies to include female subjects when assessing the long-term behavioral and motivational effects of early-life nicotine exposure.

An additional limitation concerns the use of different rat strains across experiments. Experiment 1 was conducted in male Sprague-Dawley rats, whereas Experiment 2 used male Wistar rats. Previous research has demonstrated strain-dependent differences in nicotine sensitivity and self-administration behavior, suggesting that genetic background can influence behavioral outcomes following nicotine exposure (Brower et al. 2002; Hamilton et al. 2010). Consequently, while Experiment 2 does support the conclusion that early-life nicotine exposure increases nicotine-specific motivation in male Wistar rats, it does not provide direct evidence of the long-term increases in nicotine seeking behavior following early life nicotine vapor exposure observed in Sprague-Dawley rats in Experiment 1. Future studies would benefit from employing the same rat strain across experiments, as it would allow investigators to better connect the observed results across different experiments.

Considerations in the interpretation of the PR data are also present. For example, significant increases in inactive lever presses seen in nicotine vapor-exposed adolescent rats in Experiment 1 suggest that nicotine-induced increases in psychomotor stimulation, and not increases in motivation for food reward, may be driving the increases in active lever presses for food pellets. Increases in inactive lever presses following nicotine exposure have indeed been previously reported and are attributed to nicotine’s psychomotor stimulant effect (Elliott et al. 2003; Javadi-Paydar et al. 2024). This effect will increase with higher doses of nicotine; therefore, future studies should attempt to mitigate this confound by including lower concentrations of nicotine vapor in their design. As researchers work to develop valid and reliable rodent models of e-cigarette use, it will be imperative to identify lower doses of nicotine that can maintain responding on the active manipulandum, while minimizing the effects on general locomotor stimulation, particularly when considering that adolescents are more sensitive to the rewarding effects of nicotine (Shram and Le 2010; Torres et al. 2008). Lastly, considerations regarding the PR test we conducted in Experiment 1 relate to the use of food as a reward. Nicotine has appetite-suppressing effects that may have confounded the food-deprivation induced motivation for food rewards observed. While Experiment 2 eliminated these confounds by testing rats in a PR task that utilized nicotine vapor as the reinforcer, nicotine’s relatively modest reinforcing effect creates challenges for assessing nicotine vapor seeking behavior with highly demanding response requirements. Future studies assessing the effects of nicotine on a food-based PR task should consider exploring self-administration parameters (e.g., random interval schedules of reinforcement, extended access) and employing additional controls for identified confounds, such as testing under ad lib feeding conditions.

Nicotine use disorder is the leading preventable cause of disability and death in the U.S., imposing a substantial burden on healthcare and economic systems. The ongoing and evolving nicotine vaping epidemic exacerbates this issue. While the popularity of e-cigarette use has plateaued, adolescents continue to use them at an alarming rate, with previous adolescent users continuing to use them well into adulthood. High rates of long-term use are particularly concerning, as the cumulative health effects of long-term e-cigarette use remain a mystery. Failure to identify the aggregated health risks of vaping that begins in adolescence and extends into adulthood may result in unprecedented rates of nicotine dependence, preventable death, and health care costs. Therefore, it is imperative that studies aimed at identifying the neurobiological, behavioral, social, and economic variables driving e-cigarette use throughout the lifespan, are supported and ongoing.

## Figures and Tables

**Fig. 1:**
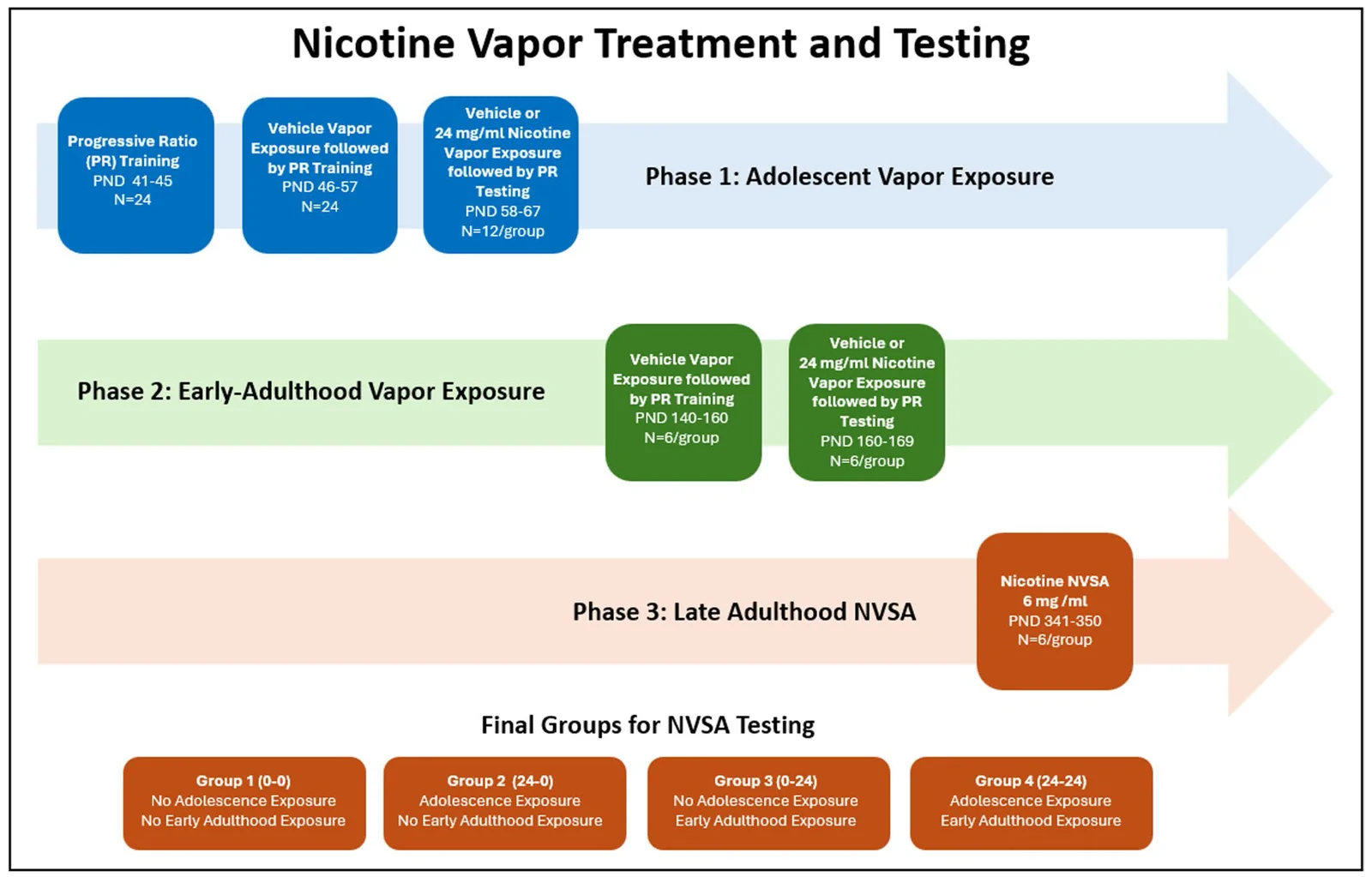
Flowchart for Experiment 1 Procedures

**Fig. 2:**
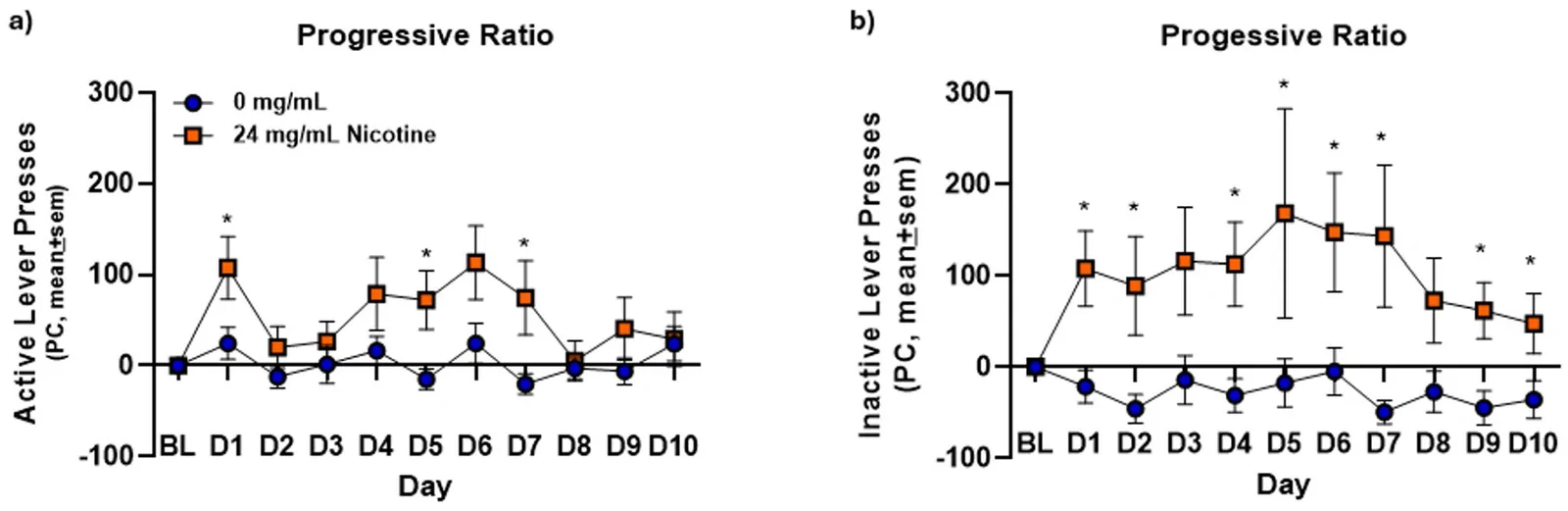
Active and inactive lever presses for food pellet delivery in PR Task during adolescent exposure. When comparing percent change in total lever presses for food pellet delivery in the PR task following adolescent nicotine vapor exposure, the 24 mg/mL nicotine group showed significant increases in active lever presses for food reward, relative to controls (**a**). Notably, 24 mg/mL of nicotine vapor exposure also caused significant increases in inactive lever presses relative to controls (**b**). *p<0.05.

**Fig. 3:**
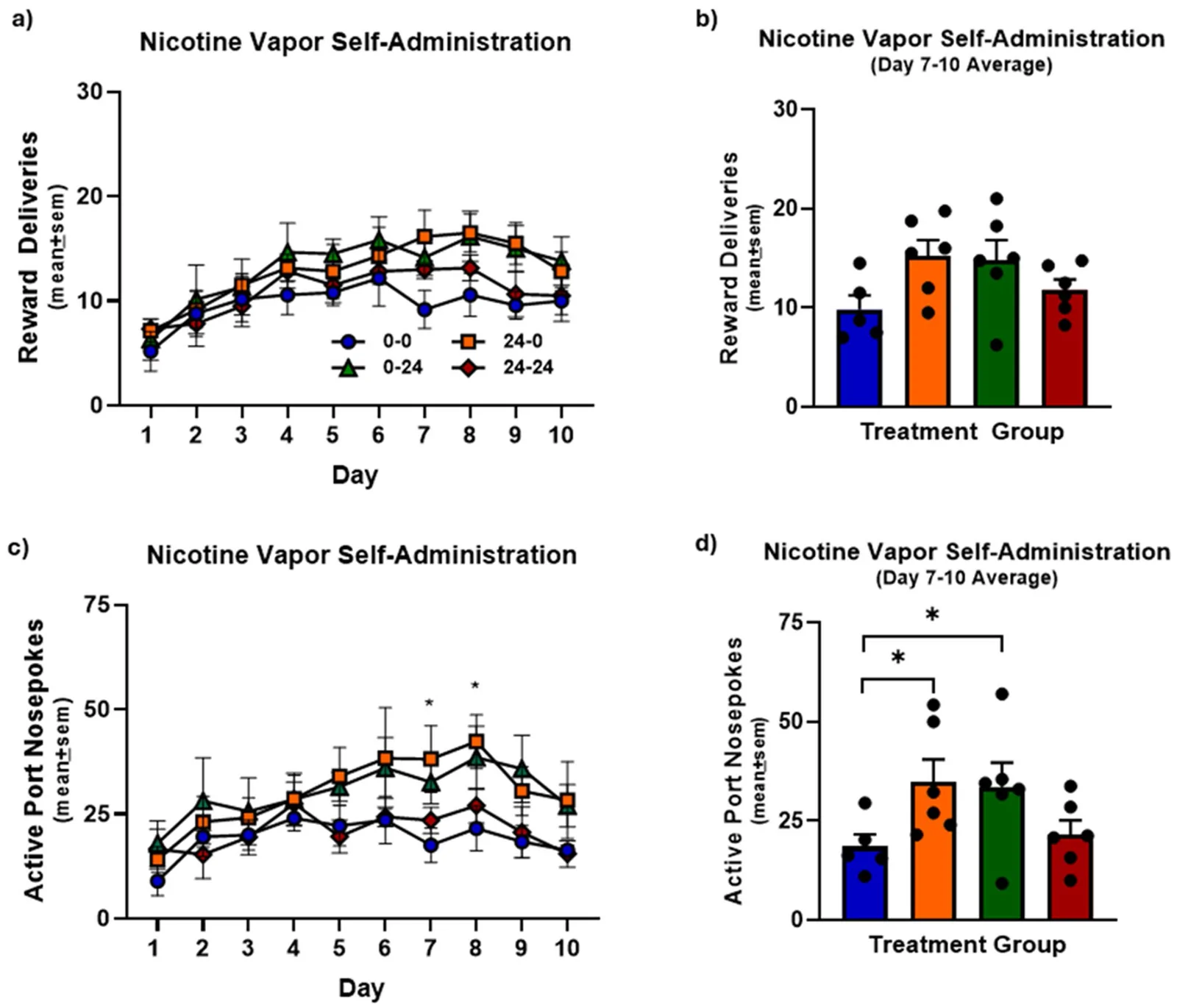
NVSA in adult rats previously exposed to nicotine. Acquisition of NVSA was observed after 6 days of training in the task. Analysis of NVSA across days 1-6 revealed no group differences in nicotine vapor deliveries (**a**) or active port nosepokes (**c**). When comparing treatment groups on days 7-10, rats exposure to nicotine during adolescence or early adulthood showed more nosepokes in the active port across NVSA days 7-10 than rats not exposed to nicotine during adolescence or early adulthood (**d**). No significant differences in vapor plume deliveries were observed between groups on days 7-10 (**b**). *p<0.05

**Fig. 4:**
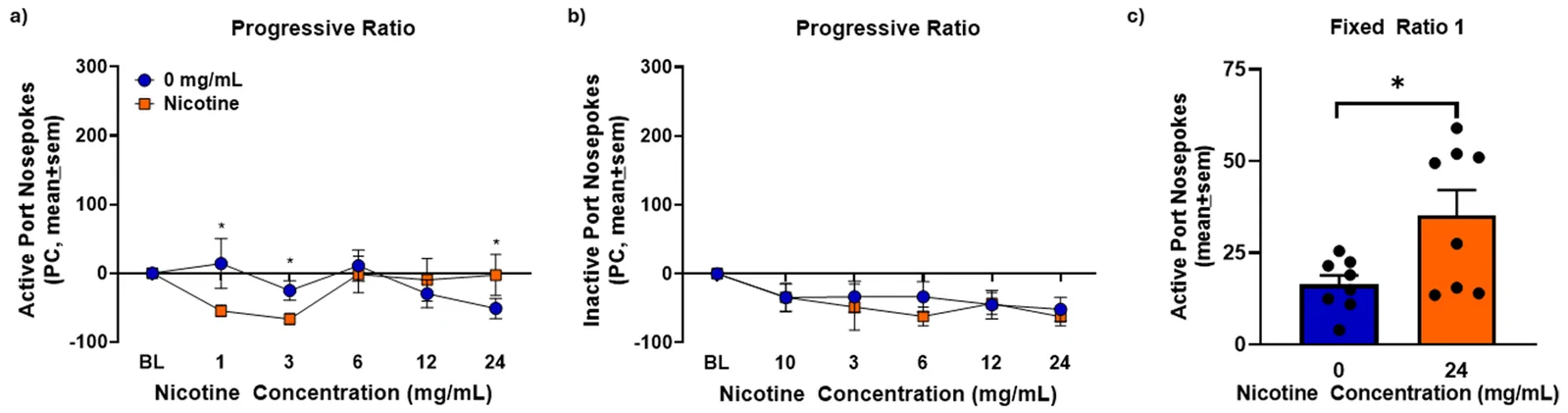
Active and inactive nosepokes for nicotine vapor delivery in PR and NVSA tasks during early adulthood. Young adult rats in the nicotine group showed significant decreases in active port nosepokes for 1 and 3 mg/mL nicotine vapor and significant increases in active port nosepokes for 24 mg/mL nicotine vapor, for relative to controls (**a**). No significant differences between vapor groups were seen for the percent change in total nosepokes in the inactive port (**b**). When testing rats on an FR1 NVSA task, rats responding for 24 mg/mL nicotine vapor displayed more nosepokes in the active port, relative to controls (**c**). *p<0.05.

## Data Availability

The raw data supporting the findings of this study are available from the corresponding author upon request.
